# Comparative genomics of eukaryotic small nucleolar RNAs reveals deep evolutionary ancestry amidst ongoing intragenomic mobility

**DOI:** 10.1186/1471-2148-12-183

**Published:** 2012-09-15

**Authors:** Marc P Hoeppner, Anthony M Poole

**Affiliations:** 1 Science for Life Laboratory and Department of Medical Biochemistry and Microbiology, Uppsala University, SE-751 23, Uppsala, Sweden; 2 School of Biological Sciences, University of Canterbury, Private Bag 4800, Christchurch 8140, New Zealand

**Keywords:** snoRNA, Last Eukaryotic Common Ancestor, Intron, Retrotransposition, Introns-first, Constrained drift

## Abstract

**Background:**

Small nucleolar (sno)RNAs are required for posttranscriptional processing and modification of ribosomal, spliceosomal and messenger RNAs. Their presence in both eukaryotes and archaea indicates that snoRNAs are evolutionarily ancient. The location of some snoRNAs within the introns of ribosomal protein genes has been suggested to belie an RNA world origin, with the exons of the earliest protein-coding genes having evolved around snoRNAs after the advent of templated protein synthesis. Alternatively, this intronic location may reflect more recent selection for coexpression of snoRNAs and ribosomal components, ensuring rRNA modification by snoRNAs during ribosome synthesis. To gain insight into the evolutionary origins of this genetic organization, we examined the antiquity of snoRNA families and the stability of their genomic location across 44 eukaryote genomes.

**Results:**

We report that dozens of snoRNA families are traceable to the Last Eukaryotic Common Ancestor (LECA), but find only weak similarities between the oldest eukaryotic snoRNAs and archaeal snoRNA-like genes. Moreover, many of these LECA snoRNAs are located within the introns of host genes independently traceable to the LECA. Comparative genomic analyses reveal the intronic location of LECA snoRNAs is not ancestral however, suggesting the pattern we observe is the result of ongoing intragenomic mobility. Analysis of human transcriptome data indicates that the primary requirement for hosting intronic snoRNAs is a broad expression profile. Consistent with ongoing mobility across broadly-expressed genes, we report a case of recent migration of a non-LECA snoRNA from the intron of a ubiquitously expressed non-LECA host gene into the introns of two LECA genes during the evolution of primates.

**Conclusions:**

Our analyses show that snoRNAs were a well-established family of RNAs at the time when eukaryotes began to diversify. While many are intronic, this association is not evolutionarily stable across the eukaryote tree; ongoing intragenomic mobility has erased signal of their ancestral gene organization, and neither introns-first nor evolved co-expression adequately explain our results. We therefore present a third model — constrained drift — whereby individual snoRNAs are intragenomically mobile and may occupy any genomic location from which expression satisfies phenotype.

## Background

**Figure 1:**
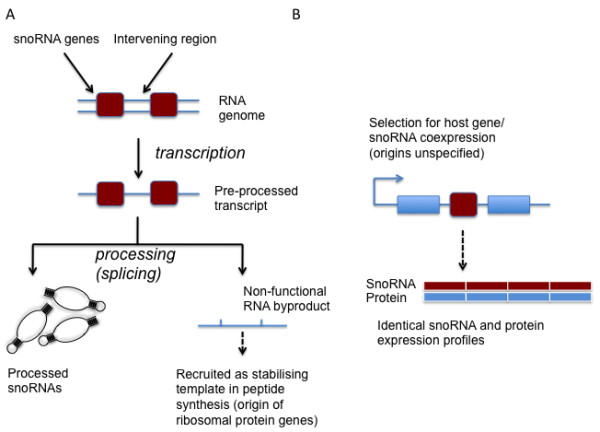
**Models accounting for the intronic location of snoRNAs. A**) The introns-first model proposes that the intronic location of snoRNAs dates back to the origin of genetically encoded protein synthesis [17]. In this model, transcription of RNA genes requires subsequent processing of precursor RNAs to release functional RNAs. Processing via splicing leads to the production of processed snoRNAs, with intervening material spliced together. This material is later coopted for stabilisation of tRNA interactions during peptide synthesis, leading to the emergence of the first mRNAs. In this model, the first proteins serve to augment RNA function, so the association between snoRNAs and ribosomal protein-coding genes is historical and evolutionarily stable. **B**) Intronic organisation of snoRNAs is the result of selection for coexpression of snoRNA and its host gene [18]. This model does not explicitly state what the ancestral condition would have been, and is therefore compatible with this organisation being ancestral (panel **A**), a more recent *de novo* origin of the snoRNA, or with this association being the result of snoRNA retrotransposition.

Small nucleolar RNAs (snoRNAs) constitute a major class of small RNA in eukaryotes. There are two broad types of snoRNA, which differ in structure and function. H/ACA snoRNAs are characterized by a double stem-loop structure, and many members of this class are involved in guiding pseudouridylation of other functional RNAs, most notably rRNA [1,2]. Most C/D snoRNAs are likewise known to be guides, directing 2'-*O*-methylation of ribose on a broad assortment of RNAs, including ribosomal RNA (rRNA) [3]. While the variety of target molecules is suggestive of an ongoing role in fine tuning or regulating a range of processes [4], including splicing [5], the antiquity of both classes of snoRNA has been established through the identification in Archaea of sno-like RNAs resembling both H/ACA and C/D family snoRNAs [6-8]. Moreover, comparative analysis of modification patterns across Bacteria, Archaea and Eukaryote rRNA indicates that some pseudouridylation and 2'-*O*-methylation modification sites may predate the diversification of the three domains of life [9,10]. However, bacterial modifications are not generated in a snoRNA-dependent manner, so while comparative genomic and experimental analyses demonstrate that snoRNAs and their associated proteins appear ubiquitous among major eukaryote and archaeal groups [11,12], there is a disconnect between the inferred antiquity of this class of RNA and conserved site modifications to rRNA. This may indicate that snoRNAs emerged following the divergence of bacteria from archaea and eukaryotes, replacing a protein enzyme-based modification system [13,14]. Alternatively, snoRNAs may have an origin in an RNA world, having coevolved with the early ribosome [15]. As part of the latter theory, it was proposed that the intronic position of snoRNAs may be related to the origin of the first mRNAs. Under this ‘introns-first’ model (Figure 1a), exons were recruited from the regions between individual snoRNAs, the latter becoming intronic following the advent of the first protein-coding genes [16,17].

While it is difficult to definitively establish the timing of emergence of snoRNAs, the introns-first model does generate testable predictions. One prediction is that these early RNA-world snoRNAs may still be housed within the introns of the protein-coding genes postulated to have evolved around them. If such an association has been preserved, host genes should be among the oldest genes, traceable to the origin of templated protein synthesis [16,17]. A strong version of this hypothesis is that this association is evolutionarily stable, such that duplication and retrotransposition have not broken up the association. An alternative view is that the intronic position of snoRNAs reflects selection for coexpression [18] (Figure 1b). This model makes no assumption as to the evolutionary and genomic mechanisms that gave rise to such an organization. A strong version of this model is that the relationship between intronic snoRNA and host gene is fixed, as per introns-first, but with one key difference: under coexpression, unless individual snoRNAs originated in the optimal genomic environment, they must have been mobile early in their evolutionary history, with selection precluding further mobility once the optimal intronic location has been reached. These hypotheses both suggest intronic position of snoRNAs has been stable across deep evolutionary timescales, so are not mutually exclusive. A third possibility is that the only constraint on snoRNA location is maintenance of an expression profile compatible with snoRNA function.

Some snoRNAs have been shown to be intragenomically mobile [19,20], with the most stunning example being reported from platypus, where a single snoRNA family was found to be present in over 40 000 copies [21]. In support of the evolutionary stability of intron-located snoRNAs, a recent study revealed that 14% of annotated snoRNAs present in more than one genome are positionally conserved across birds and mammals, and that of these 97% are intronic [22]. These observations indicate that both mobility and positional stablility of snoRNAs are observed in vertebrate genomes. However, for genomic position to be compatible with the predictions resulting from the introns-first model, there would need to be stability across the entire eukaryote tree. Given that large numbers of introns can be readily traced to the root of the eukaryote tree (i.e. the Last Eukaryotic Common Ancestor, LECA) [23], it seems reasonable to expect that a signal of positional stability, if present, should be preserved at this evolutionary depth. While the coexpression model is not mutually incompatible with introns-first, the function of snoRNAs involved in generating ribosomes means conserved intronic snoRNAs should be associated with broadly-expressed host genes, but that those host genes need not be orthologous. Across birds and mammals, previous analyses [22] indicate that the most conserved snoRNAs are encoded in broadly expressed host genes, potentially compatible with both models.

In order to better understand the evolutionary dynamics of snoRNAs, we sought to address the following questions. 1. Do any snoRNA families trace back to the LECA? 2. Do eukaryotic snoRNAs show evidence of homology with archaeal snoRNA-like sRNAs? 3. Is the intronic location of snoRNAs evolutionarily stable across the eukaryote tree? We report that dozens of snoRNA families can be traced to the LECA. However, none of these LECA snoRNA families show significant levels of similarity with archaeal sno-like RNAs, precluding firm placement of individual snoRNA families in the common ancestor of these two groups. In agreement with previous studies [23], we find numerous introns can be placed in the LECA. A subset of these introns do carry snoRNAs, but close inspection reveals that snoRNAs in equivalent conserved positions are not orthologous. This result is consistent with independent gains of unrelated snoRNAs into orthologous introns. Finally, we report that snoRNA host genes are characterised by broad expression profiles, as judged from expression patterns across 37 human tissues. We therefore conclude that these data best fit ongoing genomic mobility of snoRNAs, with selection maintaining expression patterns but not genomic location. On the basis of these results we outline a 'constrained drift' model for the evolution of snoRNA-host gene relationships.

## Results

### Placement of snoRNA families in the LECA

**Figure 2:**
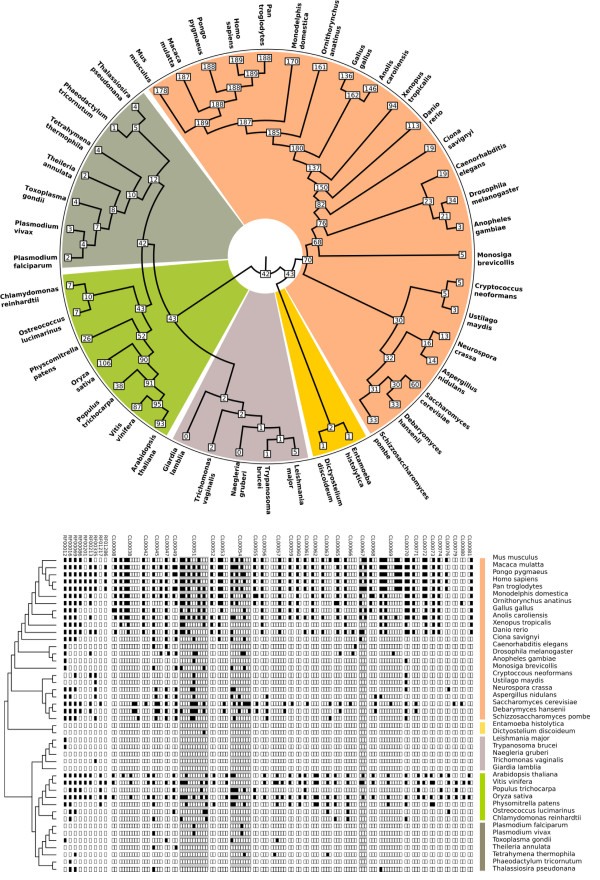
**A parsimony-based reconstruction reveals that 10 snoRNA families and 32 multi-family clans can be traced back to the Last Eukaryotic Common Ancestor (LECA). A**) Radial tree, showing numbers of Rfam families/clans at each node. **B**) The same result shown as in A, but with Rfam family and clan distribution for individual species displayed. Colors in both panels delineate the five major eukaryotic supergroups for which genomic data are currently available: Opisthokonta (fungi & metazoa), orange; Amoebozoa, yellow; Excavata, purple; Archaeplastida, green; Chromalveolata, dark green.

To establish the degree to which snoRNAs are conserved across eukaryotes, we examined the distribution of all Rfam snoRNA families identifiable across 44 eukaryote genomes (see Materials & Methods). Using Dollo parsimony, we reconstructed the pattern of snoRNA conservation across the eukaryote tree using a five supergroup phylogeny, with the root of the eukaryote tree between unikonts and bikonts [24-26]. This analysis identified 10 individual snoRNA families and 32 multi-family snoRNA clans traceable to the LECA (Figure 2; see Materials and Methods for definition of an Rfam clan**)**. Of these, 40 of 42 families/clans were C/D box snoRNAs (Additional file 1: Table S1). H/ACA snoRNAs are generally more difficult to detect owing to shorter, less well-conserved sequence motifs [27-29], which may explain the comparatively lower number of conserved families recovered by this approach.

Characterized snoRNAs are heavily biased towards plants and animals [12] (Figure 2b) so these results are dependent on the eukaryote root in Figure 2 being correctly located. However, independent attempts to locate the root of the eukaryote tree using a range of methods consistently place Opisthokonts (animals, fungi and related microbes) on the opposite side of the root from Archaeplastida [24-26,30], as per Figure 2, and minor differences in the exact placement of the root across these studies are all nevertheless compatible with the results we present here.

Rfam clans may potentially provide an additional source of information about snoRNA relationships, and could reflect family expansion through processes such as duplication [31] or retrotransposition [32]. In our analysis a clan may be placed in the LECA if two related families are treated as a single character – this could be the result of either, 1) a functional shift in one part of the eukaryote tree (orthology with a change in function), 2) hidden paralogy (with a change in function), or 3) artefactual split of a family into smaller groups so as to maintain specificity of the respective covariance models used to define a family [33]. For clans containing individual Rfam families that can each be independently placed in LECA, it may be therefore possible that the clan evolved prior to the diversification of eukaryotes, with families having evolved in the stem by duplication and divergence.

**Figure 3:**
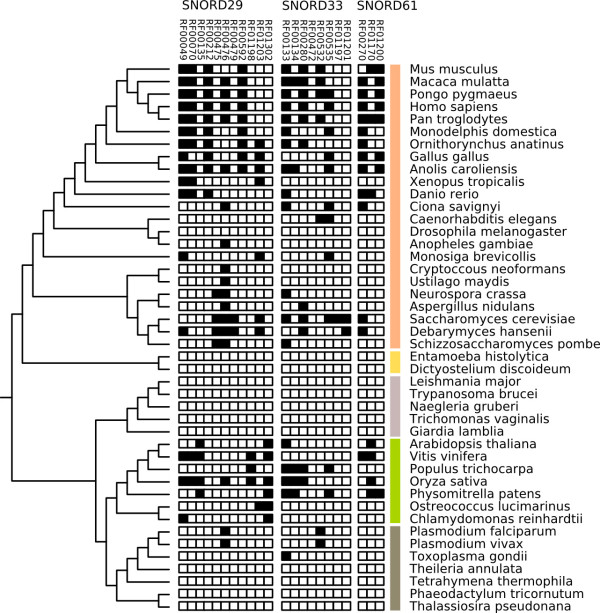
**Of 32 deeply conserved snoRNA clans, three (SNORD29, SNORD33, SNORD61) contain two or more families that can independently be placed in the LECA.** Closer inspection of predicted function (Additional file 1: Table S2) indicates that families within clans target orthologous sites, suggesting that clans are a product of the model building process in Rfam and do not reflect duplication events. Colored bars delineate eukaryote supergroups shown in Figure 2.

To assess whether clan groupings carry biological signal consistent with duplication and divergence, we examined the three clans where the constituent Rfam families could be independently traced to the LECA (Figure 3) — all three clans are comprised of C/D-box snoRNAs. For SNORD29 (CL00051), 4 of 11 snoRNA families independently traverse the eukaryote root, while for clans SNORD33 (CL00054) and SNORD61 (CL00067) respectively, 5 of 8 and 3 of 3 families predate LECA. Phylogenetic trees cannot be produced for snoRNAs at this evolutionary depth. We therefore reasoned that if individual snoRNA families traceable to LECA perform modifications at different target sites, this would be consistent with clans having evolved via duplication and divergence prior to the diversification of eukaryotes (i.e. pre-LECA). To examine this, for each clan, we took all families and established probable sites of rRNA modification for each family (Materials and Methods). We then compared our results to published experimental data on modification sites in human, yeast and *Arabidopsis*. We find support for recent expansions (several Rfam families within the SNORD29 clan are human-specific for example), but no family can be traced to duplication events predating the diversification of eukaryotes. Instead, it appears that the division of clans into families may be artefactual for these three cases, and, in the case of the SNORD61 clan, the clan likely represents the orthologous group (Additional file 1: Table S2).

### The modification targets of LECA C/D snoRNAs are also traceable to the LECA

An implicit assumption in the classification of snoRNAs into families is that each family contains members that perform the equivalent biological function across species. Consequently, if individual snoRNA families can be traced back to the LECA, we may be able to examine to what extent their function is also conserved across eukaryote evolution. However, relative to sequenced genomes/identified snoRNAs, the number of species for which modification sites for rRNA have been experimentally characterized is comparatively low [9]. We therefore sought to establish a computational means by which to predict target sites, given snoRNA and rRNA sequence data from an organism. We chose to focus on C/D snoRNAs for two reasons. First, in our initial Rfam-based analyses, the vast majority of snoRNAs putatively in LECA were C/D snoRNAs. Second, the guide sequences of C/D snoRNAs are contiguous; in contrast, H/ACA snoRNAs carry bipartite guide sequences, making computational identification non-trivial.

**Figure 4:**
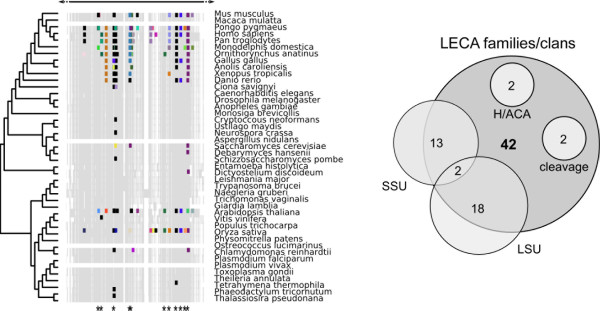
**Blast-based mapping of modification sites for all eukaryotic C/D snoRNAs in Rfam. A**) An example slice is shown, where colors correspond to individual Rfam families/clans and positions traceable to the LECA are denoted by asterisks. **B**) For SSU rRNA, we identified 23 LECA snoRNAs performing LECA-conserved 2'-*O*-methylation (13 families). For LSU rRNA, 21 LECA snoRNAs perform LECA-conserved 2'-*O*-methylation (18 families). The overlap between both data sets contained two familes. Note that, of the original 42 LECA families, four were not considered in this analysis because they either did not belong to the C/D box class (2) or are known to function exclusively in rRNA cleavage (2). Complete results are provided in supplementary material (Additional file 1: Table S3).

For each species in our dataset, we took all Rfam C/D family snoRNA entries and performed blasts against the SSU and LSU rRNA sequences from that species. This generated a blast map of potential interactions. As blast can detect similarities for reverse complements, each map detects hits equivalent to the interaction between C/D guide and cognate rRNA. To separate probable target sites from spurious blast hits, we looked for evidence of evolutionary conservation: for every putative modification site, we examined whether the distribution of hits across all species supported placement of that interaction in LECA (using parsimony). We then examined overlap between predicted LECA modification sites and Rfam families or clans previously determined (Figure 2) to be traceable to the LECA. As shown in Figure 4 there is good correspondence between predicted LECA modifications and Rfam families (Additional file 2).

As these target sites are predicted using a computational strategy, we sought to establish whether they gave reasonable correspondence to independently verified target sites and their cognate snoRNAs [34-36]. We therefore compared our set of deeply conserved modifications against known modification sites from human, yeast and *Arabidopsis*. Under the unikont/bikont rooting, *Arabidopsis* is on the other side of the root from human and yeast, so this gives us an independent means of predicting putative LECA modifications. Table 1 shows the proportion of overlap between experimentally determined modification sites and our computational predictions for *Arabidopsis*, yeast and human. Of 60 previously reported sites conserved across these three species [34-37], our comparative genomics approach fully recovers 54 (indicated by an 'X' in the respective column).

**Table 1:** **Conserved modification sites**^**a**^

	**BLAST**^**b**^			***Arabidopsis thaliana***				***Homo sapiens***				***Saccharomyces cerevisiae***			
**rRNA**	**At**	**Hs**	**Sc**	**snoRNA**	**Rfam acc**	**Rfam clan**	**Target site**^**c**^	**snoRNA**	**Rfam acc**	**Rfam clan**	**Target site**^**c**^	**snoRNA**	**Rfam acc**	**Rfam clan**	**Target site**^**c**^
LSU	X	X	X	AtU15	RF00067	**CL00045**	Am2271	U15a	RF00067	**CL00045**	Am3764	SnR13	RF01223	**CL00045**	Am2279
LSU	X		X	AtU15	RF00067	**CL00045**	Gm2278					SnR75	RF01185	**CL00045**	Gm2286
LSU	X	X	X	AtU18	RF01159	**CL00047**	Am660	U18	RF00093	**CL00047**	Am1313	U18	RF00093	**CL00047**	Am647
LSU	X	X	X	AtU24	**RF00069**		Am1451	U76	N/A		Am2350	U24	**RF00069**		Am1447
LSU	X	X	X	AtU24	**RF00069**		Cm1439	U24	RF00069		Cm2338	U24	**RF00069**		Cm1435
LSU				AtU29	RF00070	**CL00051**	Am2936	U29	RF00070	**CL00051**	Am4493	SnR71	RF00479	**CL00051**	Am2943
LSU	X	X		AtU30	RF01283	**CL00052**	Am2311	U30	RF00088	**CL00052**	Am3804				
LSU		X		AtU31	~RF00089		Gm2610	U31	RF00089	**CL00053**	Gm4166	SnR67	RF01177	**CL00053**	Gm2616
LSU	X	X	X	AtU34	~RF00147		Um1882	U34	RF00147	**CL00055**	Um2824	SnR62	RF01205	**CL00055**	Um1886
LSU	X	X	X	AtU35	RF00328	**CL00056**	Cm2949	U35	RF00211	**CL00056**	Cm4506	SnR73	RF01207	**CL00056**	Cm2956
LSU	X	X	X	AtU36a	RF01302	**CL00051**	Am2210	U36	RF00049	**CL00051**	Am3703	SnR47	RF01203	**CL00051**	Am2218
LSU	X	X		AtU37	~RF00440		Am2204	U37	RF00440		Am3697				
LSU	X	X		AtU38	RF00135	**CL00051**	Am1140	U38a	RF00212	**CL00051**	Am1858	SnR61	RF00476	**CL00051**	Am1131
LSU	X	X		AtU49	RF00337	**CL00062**	Cm1510	U49	RF00277	**CL00062**	Cm2409				
LSU	X			AtU49	RF00337	**CL00062**	Cm2869	U49	RF00277	**CL00062**	Cm4426				
LSU	X	X	X	AtU51			Am814	U51/U32a	RF00133	**CL00054**	Am1511	SnR39/59	RF01197	**CL00054**	Am805
LSU	X			AtU53,AtsnoR37			Cm2355	U53	RF00325	**CL00063**	Am3848				
LSU	X	X		AtU55	RF00358	**CL00057**	Cm1850	U55 / U39	RF00157	**CL00057**	Cm2791				
LSU	X	X	X	AtU80	RF00309	**CL00070**	Am824	U80/U77	RF00591	**CL00070**	Am1521	SnR60	RF00309	**CL00070**	Am815
LSU	X	X	X	AtU80	RF00309	**CL00070**	Um915	U80	N/A		Gm1612	SnR60	RF00309	**CL00070**	Gm906
LSU	X		X	AtsnoR1	~RF00471		Gm2781					SnR48	RF00471	**CL00066**	Gm2788
LSU	X	X		AtsnoR10	RF00353		Um2641	Nd	RF00151	CL00064	Um4197				
LSU	X	X		AtsnoR15	RF00358	**CL00057**	Cm1850	U55 / U39	RF00157	**CL00057**	Cm2791				
LSU	X	X		AtsnoR33	~RF00189		Am1861	Nd	RF00189		Am2802				
LSU	X	X		AtsnoR34	RF00133	**CL00054**	Gm2907	HB-210	RF00574		Gm4464				
LSU	X	X	X	AtsnoR35	RF01281	**CL00053**	Gm2610	U31	RF00089	**CL00053**	Gm4166	SnR67	RF01177	**CL00053**	Gm2616
LSU	X	X	X	AtsnoR37	RF00333	**CL00063**	Um2411	U52	RF00276	**CL00063**	Um3904	SnR78	RF01176	**CL00063**	Um2414
LSU	X	X	X	AtsnoR38Y	**RF00213**		Gm2805	snR38A	RF00213		Gm4362	SnR38	**RF00213**		Gm2812
LSU	X	X	X	AtsnoR39BY	~RF00055		Gm812	snR38b	RF01299	**CL00072**	Gm1509	SnR39B	RF01299	**CL00072**	Gm803
LSU	X	X		AtsnoR44	RF00357	**CL00069**	Am2316	U79	RF00152	**CL00069**	Am3809				
LSU	X	X	X	AtsnoR44	RF00357	**CL00069**	Cm2327	U74	RF00284	**CL00069**	Cm3820	SnR64	RF00509	**CL00069**	Cm2235
LSU	X	X	X	AtsnoR58Y	~RF01199		Cm674	U104	RF00289	CL00077	Cm1327	SnR58	RF01199	CL00077	Cm661
LSU	X		X	AtsnoR68Y	RF01287	**CL00079**	Am2631					SnR68	RF01235	**CL00079**	Am2637
LSU	X		X	AtsnoR69Y	RF01198	**CL00051**	Cm2938					SnR69	RF00475	**CL00051**	Cm2945
LSU	X		X	AtsnoR72Y			Am883					SnR72	N/A		Am874
LSU		X	X								Um4468	snR52			Um2921
SSU	X	X	X	AtU14			Cm416	U14	**RF00016**		Cm462	U14	**RF00016**		Cm414
SSU	X	X		AtU16	RF00358	**CL00057**	Am438	HBII-429	RF00609	**CL00073**	Am484				
SSU	X	X	X	AtU27	~RF00086		Am28	U27	**RF00086**		Am27	SnR74	**RF00086**		Am28
SSU	X	X	X	AtU36			Am621	U36a	RF00049	**CL00051**	Am668	SnR47	RF01203	**CL00051**	Am619
SSU	X	X	X	AtU43	RF00221	**CL00059**	Cm1641	U43	RF00221	**CL00059**	Cm1705	SnR70	RF01238	**CL00059**	Cm1638
SSU	X	X		AtU54	RF00206	**CL00008**	Gm597	U54	RF00206	**CL00008**	Gm644				
SSU	X	X		AtU56			Cm471	U56	RF00275		Cm517				
SSU	X	X		AtU61	RF01170	**CL00067**	Um1381	U61	RF00270	**CL00067**	Um1442				
SSU	X	X		AtsnoR14	RF01280	**CL00076**	Um1232	HBII-55	RF00610	**CL00076**	Um1288				
SSU	X	X		AtsnoR15	RF00358	**CL00057**	Am438	HBII-429	RF00609	**CL00073**	Am484				
SSU	X	X		AtsnoR17	RF01289		Am466	U45a			Am512				
SSU	X	X		AtsnoR18			Am162	U44	RF00279		Am166				
SSU	X	X	X	AtsnoR19	RF00054	**CL00049**	Gm1431	U25	RF00054	**CL00049**	Gm1490	SnR56	RF01188	**CL00049**	Gm1427
SSU	X	X	X	AtsnoR21			Gm1272	U32	RF00133	**CL00054**	Gm1328	SnR40	RF01201	**CL00054**	Gm1269
SSU	X	X	X	AtsnoR23	RF00350		Am1754	Nd			Am1850	Nd			Am1779
SSU	X	X		AtsnoR30	RF00046	**CL00073**	Gm390	HBII-429	RF00609	**CL00073**	Gm436				
SSU	X	X		AtsnoR32	RF00330		Am1327	snR53	RF00338	**CL00080**	Am1383				
SSU	X	X	X	AtsnoR34	RF00133	**CL00054**	Um1270	U33	RF00133	**CL00054**	Um1326	SnR55	RF00472	**CL00054**	Um1267
SSU	X	X	X	AtsnoR41Y	~RF00153		Am543	U62a	RF00153	**CL00068**	Am590	SnR41	RF01218	**CL00068**	Am541
SSU	X	X	X	AtsnoR53Y	RF01279	**CL00080**	Am799	HBII-429	RF00609	**CL00073**		SnR53	RF00338	**CL00080**	Am796
SSU	X	X		AtsnoR58			Gm391	SNORD100	**RF00609**		Gm436				
SSU	X	X	X	AtsnoR59	~RF00273		Am975	U59	RF00273	**CL00065**	Am1031	SnR54	RF00473	**CL00065**	Am975
SSU	X	X	X	AtsnoR77Y			Um580	HBII-135	RF00571	**CL00057**	Um627	SnR77	RF01181	**CL00057**	Um578
SSU	X	X	X	U33			Um1270	U33	RF00133	**CL00054**	Um1326	SnR55	RF00472	**CL00054**	

^a^Families/clans traceable to LECA based on distribution and parsimony (bold).

^b^Blast-mapped hits (X) show agreements with analysis presented in Figures 4 and 5.

^c^Target site data derived from '3D ribosomal RNA modification database' [37].

**Figure 5:**
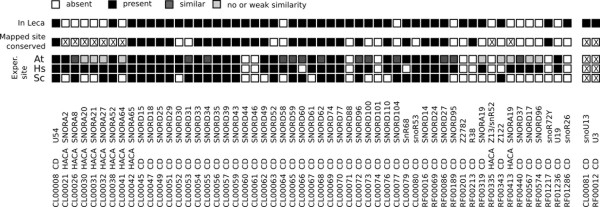
**Comparison of snoRNAs traceable to LECA (Figure**1**) and those experimentally characterised 2'-*O*-methylation sites attributable to LECA based on conservation between *Arabidopsis thaliana *(At) and at least one of *Homo sapiens *(Hs) and *Saccharomyces cerevisiae *(Sc).** Using these independent lines of evidence, 25 families can be placed in the LECA. Plant snoRNAs are not always recognized by Rfam models for the equivalent animal snoRNA, suggesting that Rfam coverage is currently incomplete (Figure 4; light-grey boxes). Two of the snoRNAs listed here (U3, U13) do not function as modification guides and therefore cannot be mapped to existing sites.

To gauge the reliability of the three types of data (snoRNA genes, blast-mapped modification sites and independently determined sites), we compared the results of all three methods (Figure 5). The intersection between the 42 LECA snoRNAs, 28 LECA blast-mapped sites and 37 independently-mapped LECA modification sites is 25. We note however that this is an underestimate: two LECA snoRNA families (snoU13 and U3) are involved exclusively in rRNA cleavage but not 2'-*O*-methylation, and two further putative LECA-snoRNAs belong to the H/ACA class (which we did not map against rRNA) - so are automatically excluded from this intersect for this reason. In summary, a stringent, total evidence-based approach (Figure 5) identifies a minimum of 25 modification guide C/D snoRNA families with conserved function traceable to the root of the eukaryote tree, under the unikont/bikont topology.

### Some LECA snoRNAs have known archaeal counterparts

**Figure 6:**
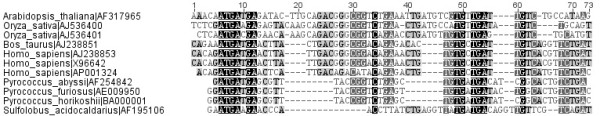
**Alignment of eukaryote SNORD43 with archaeal sR12 sequences from *Pyrococcus *and *Sulfolobus *indicates detectable sequence similarity, suggestive of common ancestry.** The alignment does not yield a conserved secondary structure, and therefore the possibility of convergence cannot be ruled out.

Archaea and eukaryotes both carry an equivalent H/ACA and C/D RNA-based guide machinery, with a conserved set of associated proteins [11,12]. However, it is not trivial to identify archaeal and eukaryote RNA counterparts using sequence data alone. We therefore took the alternative approach of asking whether any archaeal sno-like sRNAs are responsible for modification at a position in archaeal rRNA equivalent to conserved eukaryotic rRNA modifications. This identified only one case, archaeal sR12, found in both crenarchaea and euryarchaea, which had previously been predicted by Gaspin and co-workers to guide 2'-*O*-methylation at an equivalent site to yeast snR70 [6]. Our analysis (Figure 2) placed the snoRD43/snR70 clan (CL00059) in LECA. In order to examine possible homology, we generated a multiple sequence alignment of members of the CL00059 clans and archaeal sR12 sequences using an alignment algorithm optimised for non-coding RNA [38], and sought to establish whether secondary structure was also conserved, using alifold from the Vienna package [39]. The alignment shown in Figure 6 shows similarities in the guide sequence, but we find no clear indication of structural conservation. Consequently, while these findings are consistent with SNORD43 & sR12 being orthologous (and therefore predating the archaeal-eukaryotic divergence), we cannot rule out that possibility that these sequence similarities are a result of convergence given selection for the underlying function. Gaspin and colleagues reported 4 additional cases of modification equivalence, three of which correspond to eukaryotic snoRNA families in our Rfam dataset. However, these could either not be placed in LECA (snR52) or exhibited limited sequence similarity, precluding clear inference of common ancestry (snoRD60/sR36 and snoRD96/sR11).

### Testing the introns first hypothesis for the origin of mRNA

Available data are consistent with snoRNA-based rRNA processing/modification being present in the common ancestor of archaea and eukaryotes [11,12]. However, an earlier RNA world origin has also been suggested [17,40]. Consistent with this, a small number of experimentally-characterized 2'-*O-*methylation and pseudouridylation sites on rRNA are conserved across all three domains of life [9], and we note that other RNA modifications likewise appear to have evolved prior to divergence of the three domains [10]. However, an alternative view is that the ancestral system for 2'-*O-*methylation and pseudouridylation was protein-enzyme based as in extant bacterial lineages, with the snoRNA-based system evolving in response to a need for more extensive modifications in archaea and eukaryotes [13,14]. It is not possible to directly test these alternative scenarios, both of which are plausible. However, one aspect of the 'snoRNAs-ancient' view is amenable to testing through comparative genomics. Under the introns-first model, the intronic location of snoRNAs is an ancestral state, predating the origin of genetically-encoded protein synthesis. Briefly, the ancestral state is hypothesized to be RNA genes linked on chromosomes; the intervening spaces between these RNAs is later coopted into the role of messenger RNA. This model assumes that processing of individual snoRNAs from larger polycistronic RNA transcripts occurred via a proto-spliceosome [16,17] (Figure 1a). Under this model, these intervening regions are coopted as the first proto-exons. Consistent with this view, many snoRNAs are housed in the introns of ribosomal protein genes, particularly in vertebrate genomes [22]. Moreover, ribosomal protein genes are one of the most ancient classes of gene in the cell [41], and, significantly, an evolutionary history dominated by vertical descent is apparent for this class of genes [42]. While any scenario for very early evolution is by nature speculative, introns-first predicts that the intronic position of snoRNAs is ancestral. If the intronic location of any snoRNA is conserved across the eukaryote tree, such a pattern would be consistent with introns-first (with the corollary that introns have been lost/reduced to self-splicing forms in archaea and bacteria; of note, complete intron loss has been documented in the *Hemiselmis andersenii* nucleomorph [43]. We therefore sought to establish whether intronic snoRNAs are positionally conserved across eukaryotes.

We first screened our dataset of 44 eukaryote genomes for introns containing annotated snoRNAs. Our screen of eukaryote genomes yielded a set of 1782 host genes carrying intronic snoRNAs. Of these, 1091 of the host genes could be placed in LECA on distribution (Additional file 1: Figure S1; Additional file 3).

**Figure 7:**
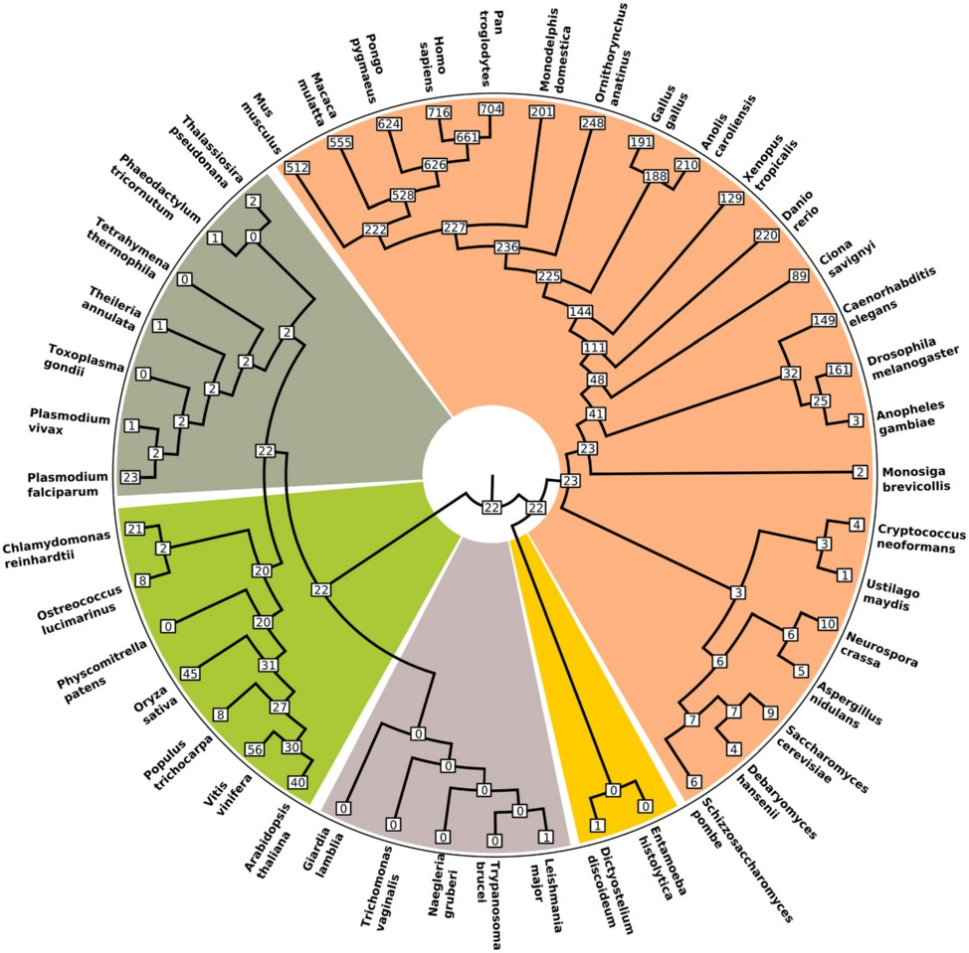
**Positional equivalence of intronic snoRNAs in LECA genes.** We binned snoRNA-carrying introns into discrete loci based on protein-based alignments of their respective genes. The results suggest that, on parsimony, 22 loci are traceable to the LECA. However, in no case were we able to establish homology of snoRNAs across supergroups (based on sequence, structure or modification site), suggesting independent gain rather than deep ancestry (see text). Colors are as per Figure 2.

To establish if individual introns within the cohort of LECA host genes could also be traced to LECA, we reconstructed intron presence in LECA using parsimony (see methods). This yielded 9117/98871 (7.6%) orthologous intron loci that can be traced to LECA (Additional file 1: Figure S2), in broad agreement with other studies indicating high numbers of introns can be traced to LECA [23]. For any intron carrying a snoRNA, we next independently compared intron ancestry with snoRNA intron occupancy across the eukaryote tree. This yielded 22 orthologous LECA introns that also carry a snoRNA (Figure 7). Finally, we examined Rfam family constituency for all 22 cases across all eukaryote genomes. We reasoned that if all positionally equivalent snoRNAs were from the same family, this would provide an initial indicator of snoRNA orthology.

Strikingly, we find no evidence for common ancestry for any of these 22 cases, based on Rfam family membership (Table 2), and in many cases the snoRNAs are not even of the same type (i.e. H/ACA or C/D), clearly precluding homology. This pattern, while precluding the placement of intronic snoRNAs in LECA, does not in itself indicate snoRNA mobility. For the 22 cases of non-orthologous snoRNA occupancy in Figure 7, we therefore examined snoRNA distribution more closely in an attempt to distinguish ancestral from derived snoRNA occupancy among orthologous introns. As shown in Table 2 none of the putative LECA-intronic snoRNAs can be claimed to trace to the LECA, being restricted for the most part to a few species. Manual inspection of alignments and sequence features for these equivalent intronic snoRNAs from the same class did not reveal any cases of homology. We therefore conclude that these data do not support the evolutionary stability of intronic snoRNAs, predicted under the introns-first hypothesis.

**Table 2:** snoRNAs in deeply conserved introns

**Group**	**snoRNA**	**Species**	**Supergroup**	**class**	**Cross-Supergroup comparison**
ENSG00000221983	H06I04.8	*C. elegans*	Opisthokonts	CD	No evidence for homology
	RF00332	*A. thaliana*	Archaeplastida	CD	
ENSG00000142541	RF00133	Mammals	Opisthokonts	CD	Unrelated classes //
	FBgn0260002	*D. melanogaster*	Opisthokonts	HACA	No evidence for homology
	OssnoR30	*O. sativa*	Archaeplastida	CD	
ENSG00000143947	H06I04.8	*C. elegans*	Opisthokonts	HACA	Unrelated classes
	AtsnoR91	*A. thaliana*	Archaeplastida	CD	
	RF00332	*O. sativa*	Archaeplastida	CD	
ENSG00000213516	RF00270	*Vertebrates*	Opisthokonts	CD	Unrelated classes
	RF01287	*V. vinifera*	Archaeplastida	HACA	
ENSG00000204628	RF00270	Vertebrates	Opisthokonts	CD	No evidence for homology
	Pfa_snoR_05	*P. falciparum*	Chromalveolata	CD	
ENSG00000241343	Fbgn0065055	*D. melanogaster*	Opisthokonts	HACA	Unrelated classes
	RF00202	*O. sativa*	Archaeplastida	CD	
	RF00147	*P. trichocarpa*	Archaeplastida	CD	
ENSG00000147274*	RF00270	*Vertebrates*	Opisthokonts	CD	Unrelated classes
	RF01287	*V. vinifera*	Archaeplastida	HACA	
ENSG00000149273	RF00067	Vertebrates	Opisthokonts	CD	Unrelated classes
	RF01432	*O. sativa*	Archaeplastida	HACA	
ENSG00000170515	Fbgn0082994	*D. melanogaster*	Opisthokonts	HACA	Unrelated classes
	RF00218	*A. thaliana*	Archaeplastida	CD	
	RF00218	*V. vinifera*	Archaeplastida	CD	
ENSG00000165502	Fbgn0065055	*D. melanogaster*	Opisthokonts	HACA	Unrelated classes
	RF00202	*O. sativa*	Archaeplastida	CD	
ENSG00000232055**	RF00150	*M. domestica*	Opisthokonts	CD	No evidence for homology
	RF00145	*O. sativa*	Archaeplastida	CD	
	RF00067	*O. sativa*	Archaeplastida	CD	
ENSG00000215472	RF00151	Vertebrates	Opisthokonts	CD	No evidence for homology
	RF00067	*P. vivax*	Chromalveolata	CD	
	Pfa_snoR_21	*P. falciparum*	Chromalveolata	CD	
	RF00067	*T. annulata*	Chromalveolata	CD	
ENSG00000215472	RF00151	Vertebrates	Opisthokonts	CD	Unrelated classes
	Fbgn0086602	*D. melanogaster*	Opisthokonts	HACA	No evidence for homology
	Fbgn0086668	*D. melanogaster*	Opisthokonts	HACA	
	Fbgn0083013	*D. melanogaster*	Opisthokonts	HACA	
	RF001231	*A. thaliana*	Archaeplastida	HACA	
	RF001231	*V. vinifera*	Archaeplastida	HACA	
	RF001231	*O. sativa*	Archaeplastida	HACA	
ENSG00000145592	RF00577	*D. rerio*	Opisthokonts	CD	No evidence for homology
	RF00200	*O. sativa*	Archaeplastida	CD	
	RF00267	*O. sativa*	Archaeplastida	CD	
	RF00093	*O. sativa*	Archaeplastida	CD	
ENSG00000109971	RF00016	*D. rerio*	Opisthokonts	CD	Unrelated classes
					No evidence for homology
	RF00482	*O. sativa*	Archaeplastida	HACA	
	CL00053	*O. sativa*	Archaeplastida	CD	
	AtsnoR93	*A. thaliana*	Archaeplastida	HACA	

* Set of 7 human paralogues.

** Set of 2 human paralogues.

### LECA genes hosting intronic snoRNAs are broadly-expressed

**Figure 8:**
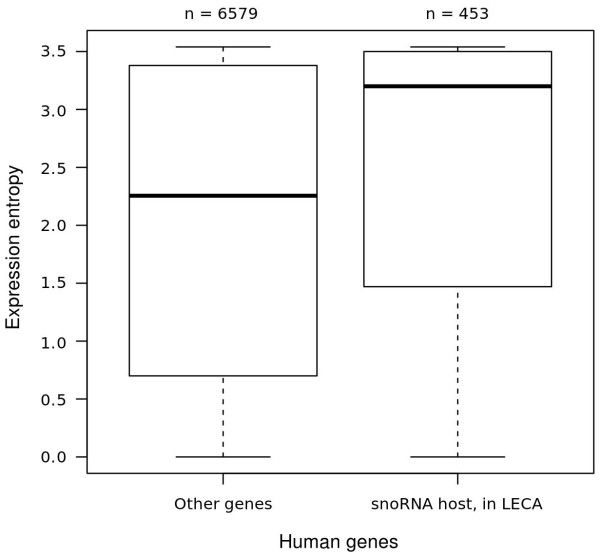
**Expression entropy (breadth of expression across tissues) based on data from the Norvatis transcriptome atlas **[45]**shows that deeply conserved, snoRNA carrying host genes are generally more broadly expressed than non-carrying genes.** On the backdrop of a requirement for broad snoRNA expression, this may suggest a selective advantage for this association. A total of 7022 genes were considered, based on usable expression data and reliable correlation to EnsEMBL genes ids in release 58.

While our results demonstrate that intronic location of snoRNAs does not trace to the LECA, it is striking that 61% (1091/1782) of snoRNA-containing host genes in our study can themselves be placed in the LECA (Additional file 1: Figure S1). We were therefore interested to know what features of these broadly-distributed genes might make them an ideal location for intronic snoRNAs. As rRNA function is essential, modification of rRNA should also be required in all cell types in multicellular organisms. We may therefore expect that the main requirement for host genes is that they should be ubiquitously expressed. We previously showed that for positionally conserved intronic snoRNAs in the bird-mammal ancestor, intronic snoRNA-containing genes were broadly expressed, and fundamental processes such as translation were significantly overrepresented amongst this cohort, whereas intronic snoRNAs with a shallower evolutionary association with their host gene (traceable to the primate ancestor) were not overrepresented among broadly-expressed genes [22]. More generally, ‘older’ genes are known to be more broadly expressed across a range of tissues in vertebrates [44]. We therefore examined the expression pattern of the entire set of 1091 LECA host genes using human expression data [45]. As shown in Figure 8, the distribution of expression entropies of human genes shows that genes hosting an intronic snoRNA are more broadly expressed (high entropy) than non-host genes (Mann–Whitney-U, p < 0.005). We conclude that the preference for integration into the introns of ancient (LECA) host genes is a consequence of these genes being broadly expressed. Such host genes are compatible with the requirement for broad expression of snoRNAs.

### Recent migration of a snoRNA into the intron of a ribosomal protein gene

**Figure 9:**
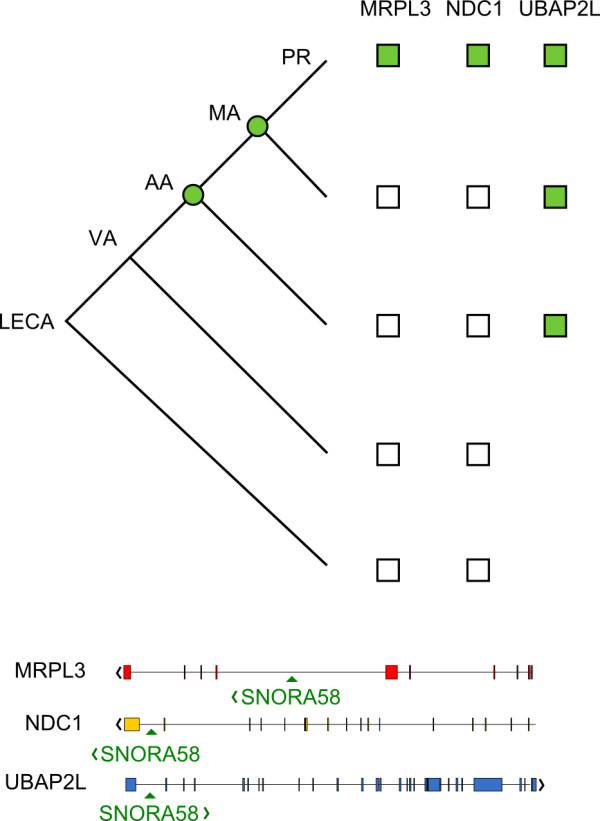
**Recent gain of a snoRNA in a ribosomal protein of mitochondrial origin.** The tree (top graphic) indicates depth of association of SNORA58 with its host introns. Circles indicate the distribution of SNORA58, while squares indicate distribution of the protein. MRPL3 [47-49] and NDC1 [46] can be traced to the LECA, while UBAP2L is traceable to the vertebrate ancestor (data not shown). Expression data [51] indicate all three host genes are widely expressed in amniotes (Additional file 4). Grey squares indicate SNORA58 presence in the intron. The bottom graphic shows the position of SNORA58 in the introns of each gene is indicated by arrowheads. Gene orientation is indicated by < and >. The gene structure in the figure is from human. LECA—Last Eukaryotic Common Ancestor, VA—Vertebrate Ancestor, MA—Mammalian Ancestor, PR – Primate ancestor.

As none of the non-homologous 22 snoRNAs identified in LECA introns could be definitively attributed to derived mobility-associated gain (to the exclusion of secondary losses), we combed our dataset for intronic snoRNAs that could be unambiguously attributed to gain. Figure 9 shows one such example. SNORA58 is an intronic snoRNA, the ancestral position of which is within the ubiquitin associated protein 2-like gene, UBAP2L. Both UBAP2L and SNORA58 trace to the amniote (mammal/bird) ancestor, but, prior to the divergence of primates, SNORA58 has expanded into the introns of two LECA genes, MRPL3 and NDC1. NDC1 is a nuclear pore complex constituent that traces to the LECA [46], whereas MRPL3 is a nuclear-encoded constituent of the mitochondrial ribosome, and ultimately derives from the bacterial ancestor of the mitochondrion [47-49]. Further, the introns in the MRPL3 gene derive from intron gain [50] and, in the case of SNORA58, the host intron is positionally-conserved across opisthokonts and plants. We therefore conclude that SNORA58 has moved into genes traceable to LECA, and that, in the case of MRPL3, this is an irrefutable case of gain of both intron and intronic snoRNA.

## Discussion

Under the RNA world hypothesis, that genetically-encoded proteins and DNA genomes were preceded by functional RNAs and RNA genomes, it has previously been suggested that many RNA-based processes may directly trace back to this early stage in the evolution of life. Such arguments have largely been made on the basis of comparisons of functional RNAs [40,52], though fewer than 1% of known RNA families show evidence of deep evolutionary ancestry [53]. For snoRNAs, while a compelling case can be made for an RNA-world origin [17,40], it is equally possible that this class of RNA has a more recent origin [13,14]. In so far as eukaryotic snoRNAs have counterparts in archaea, and given that, in both domains, these RNAs interact with a common set of associated proteins [11,12], the origin of snoRNAs in the common ancestor of both domains appears likely [6].

We aimed to specifically examine the antiquity of snoRNAs using comparative genomics. Our results confirm that individual snoRNA families can be placed in the Last Eukaryotic Common Ancestor. Using a conservative total-evidence approach, incorporating Rfam family conservation, a blast-based comparative genomics approach to identifying target modification sites, and comparison with experimentally-mapped sites, we report that a minimum of 25 C/D type snoRNAs can be placed in the LECA.

Based on a conservative estimate of snoRNAs in LECA, we found three LECA snoRNAs for which a positionally-conserved modification was present in archaea. While the archaeal counterpart was known for all these cases, we deemed the sequence and secondary structure similarities across domains to be insufficient for an inference of homology to be made. While the equivalence of the modification-sites is striking, our analysis was unable to provide stronger evidence in favour of a common origin for individual RNA families. It may simply be the case that, given that snoRNAs are short, evolutionary signal, if it did exist, has been erased over such timescales.

Finally, we aimed to establish whether the intronic location of snoRNAs is consistent with snoRNA gene mobility, with selection favouring colonisation of introns in genes with broad gene expression, or whether position is evolutionarily conserved. We find that, despite intron position being conserved across the eukaryote tree (suggestive of an intron-rich LECA), none of the snoRNAs residing in these putative LECA introns are positionally stable at this evolutionary depth. A specific prediction of the introns-first model is that snoRNAs are found in the introns of the most deeply-conserved protein-coding genes. Evolutionary signal consistent with this model requires the intron, the snoRNA associated with that intron, and the position of the snoRNA within the intron to all be traceable to the LECA. Our analysis therefore rejects this strict version of the introns-first hypothesis. As per the introns-early vs introns-late debate [16,54,55], alternative versions of introns-first are possible, and the snoRNA mobility we observe is not in itself incompatible with an RNA-world origin. However, if, as our data suggest, all trace of any patterns compatible with an early origin have been lost (if they were present at all), an early origin necessarily remains speculative.

**Figure 10:**
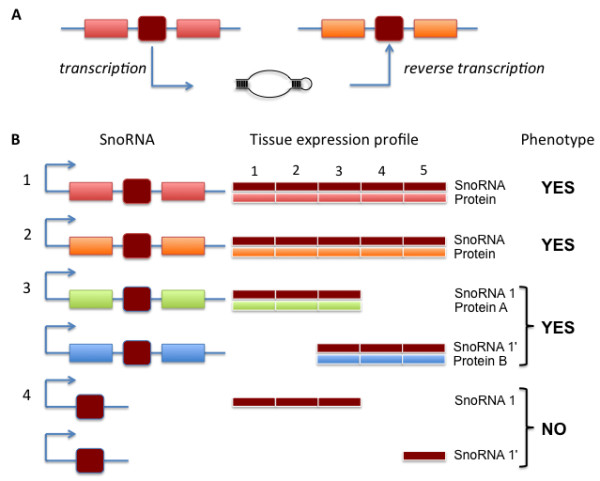
**SnoRNA evolution by constrained drift.** In this model, snoRNAs are genomically mobile and may vary in copy number (through processes of retrotransposition or DNA-level duplication). **A**) An intronic snoRNA (dark red) may be integrated into a new genomic location (orange gene) through retrotransposition. The original copy is retained (light red gene) leading to a copy number increase. **B**) The genomic location (and copy-number) of snoRNAs is constrained only by the requirement for phenotype to be satisfied, and a range of genomic organisations may satisfy phenotype. Within these bounds, multiple genomic architectures may generate the same phenotypic outcome, so architecture and copy number may be free to drift within these bounds. The top three expression profiles satisfy phenotype, and are therefore viable. Cases 1 & 2 show different host genes with equivalent expression profiles, whereas case 3 exhibits subfunctionalisation, where combined expression from two host genes, each with limited expression profiles, satisfies the required phenotypic expression profile through expression of the equivalent snoRNA. Profile 4 shows two (non-intronic) copies of a snoRNA gene, where the full expression profile is not achieved. An individual with such an expression profile would be eliminated from the population under constrained drift. SnoRNA genes (black squares); exons (rectangles); snoRNA expression profiles (thin black bars). Primes (') indicate a functionally equivalent snoRNA copy. Note that proteins A & B need not be evolutionarily related.

We suggest that our results may instead better fit an alternative evolutionary model which we call 'constrained drift' (Figure 10). In this model, individual snoRNAs can migrate to different genomic locations, provided the overall expression profile is preserved. This model is compatible with a range of genetic organisations, including a single snoRNA expressed from a ubiquitously-expressed host gene, subfunctionalised snoRNAs expressed from host genes with compatible, non-overlapping expression profiles, and independent snoRNA genes with either ubiquitous or subfunctionalised expression profiles. As phenotype is satisfied by all four cases, we predict that genomic location and organisation should be free to drift and is selectively constrained only by the requirement that a ubiquitous expression profile is maintained.

Our analysis of expression profiles of human genes bearing intronic snoRNAs (Figure 8) indicates that snoRNA-containing genes have a broad expression profile. However, these expression data do not enable us to assess individual expression profiles of snoRNAs, and we have not examined snoRNA paralogy, both of which would need to be examined to fully determine whether one of the gene organisation patterns described in Figure 10 dominates. High-throughput expression data may soon enable us to examine this question in more detail.

## Conclusions

Using an evolutionary analysis, we have shown that the snoRNA apparatus was well-established in the LECA. While numerous snoRNAs can be traced to the LECA, the intronic location of individual snoRNAs is not stable over large evolutionary timescales, with no evidence for positional conservation traceable to the LECA. The data presented here fit a model of ongoing mobility over shorter evolutionary timescales [19,32], with natural selection acting to constrain drift. As shown in Figure 10, some genomic locations may be selected against if function is compromised. However, a more extreme drift model is plausible, since this role for selection assumes that all modifications are under strong selection, a contention which has not been demonstrated. In light of experimental studies showing weak observable phenotype associated with individual snoRNA knockouts [56-58], it will be interesting to establish to what extent the modification of rRNAs by snoRNAs is governed by drift versus selection. For the set of most deeply conserved snoRNAs identified here, we suspect that constrained drift best accounts for their genomic evolution. However, for snoRNAs with a more recent evolutionary history the 'extreme' version of the drift model provides a valuable null hypothesis, which may be particularly helpful in assessing the significance of knockout studies [59-61]. While it is possible that snoRNAs have expanded into novel functions [5], it is notable that no other examples have been reported. Rather than representing a cohort of novel functional RNAs, some snoRNAs housed in narrowly-expressed host genes [22] may well be neutral, rather than the outcome of neofunctionalisation (Figure 10). Combining comparative analyses, large-scale expression data and knockout phenotypes will be required to resolve this question.

## Methods

### Genome dataset assembly

We built a custom database, based on a local installation of the EnsEMBL PanCompara database [62]. Additional genome data, including cDNA coordinates and snoRNA annotations, were obtained from one of two sources: where available, these were downloaded directly from the Ensembl database, release 58 [63,64], through a Ruby-based API [65]. For Non-EnsEMBL species, genomes were downloaded from GenBank and parsed into a custom database using bio-ruby version 1.2 [66]. A full list of species is shown in Additional file 1: Table S3. Orthologous genes (identified as described below) from these genomes were subsequently merged into the comparative database.

### SnoRNA dataset

Our snoRNA dataset derives from release 10 of the Rfam database [67]. Release 10 can be downloaded from the Rfam website (rfam.sanger.ac.uk). Rfam release 10 allows for RNA families which may perform distinct functions yet share sequence or structural similarity to be grouped into higher units, called clans. Clans are a pragmatic approach to ensuring homologous snoRNAs are not artefactually split into discrete families, and are described in detail elsewhere [67]. Rfam clans are representative of homology, but do not distinguish between orthologous and paralogous relationships. As we used both Rfam clans and families in our analysis, we assessed clans for evidence of paralogy, as described in the section, ‘Identification of Putative Modification Sites via Comparative Analyses’.

The genomic location of snoRNAs was determined in several complementary ways. First, existing annotations were imported from the EnsEMBL database [63,64]. These annotations are based either on experimentally verified sequences and derived from specialist databases (including Flybase [68] and Wormbase [69]) or stem from computational predictions using Rfam covariance models (most EnsEMBL genomes) [68]. The latter are built from curated and thresholded seed alignments combined with information on conserved structural motifs, as described in Rfam documentation [67]. Each Rfam family corresponds to a group of significantly similar sequences [67] and was used in our study to establish snoRNA homology across species.

We then checked the literature for additional snoRNAs not yet included in Rfam, and added these to our data set (Additional file 1: Table S4). Genomic coordinates were determined using BLAT [70], allowing for full-length matches with fewer than 2 substitutions. Where possible, sequences were assigned to an existing Rfam family.

We also used the (unmodified) Rfam annotation script (available from the Rfam FTP server at ftp://ftp.sanger.ac.uk/pub/databases/Rfam/) to identify additional snoRNA candidates across all genomes (unless such annotations were readily available from EnsEMBL) and added these to the final dataset. A full list of annotations with genomic coordinates is available from the authors upon request.

### Assessing host gene orthology

Orthologous groups of snoRNA-bearing host genes were constructed in two steps using human genes as seed. First, for all EnsEMBL genomes, orthologs were obtained directly from the EnsEMBL database [71]. For non-EnsEMBL genomes (Additional file 1: Table S3), putative orthologs were identified using the InParanoid algorithm [72]. Each group of orthologs carrying at least one snoRNA (or snoRNA candidate) was subsequently aligned on the protein level using ProbCons [73], yielding a total of 1782 alignments to be used in the reconstruction of ancestral states for snoRNA and intron loci (below). A full list of groups and gene accession numbers is available upon request.

### Identification of putative modification sites via comparative analyses

We reasoned that since C/D family snoRNAs possess linear and well-defined guide sequences, it should be possible to identify putative interactions using conserved sequence complementarity between snoRNA and target RNA. Putative ribosomal RNA targets of methylation-guide (C/D) snoRNAs (including Infernal-derived candidates – see above) were therefore predicted through a comparative approach that utilised blastn from the Blast package [74]. To ensure specificity, search parameters were set to a word-size (W) of 5 and gap open (G) and gap extension (E) penalties of 50. Curated alignments of small- and large-subunit ribosomal RNA sequences were obtained from the SILVA database [75] (Additional file 1: Table S3). Ribosomal RNA sequences for *Arabidopsis thalianaHomo sapiens* and *Saccharomyces cerevisiae* corresponding to those used in specialist snoRNA databases [34-36] and previous publications [9] were added to the alignment using the profile alignment option in ClustalW [76].

All putative rRNA-C/DsnoRNA interactions were binned into loci based on their center position (+/− 2 nucleotides). Loci restricted to a single species were discarded. We then reconstructed the ancestral state for all modification sites across species for families that we previously identified as likely LECA-candidates. Only those candidate sites whose phylogenetic distribution was compatible with presence in LECA were considered, resulting in a total of 40 deeply conserved sites. To gauge the accuracy of this approach, we compared predicted, conserved sites with previously reported methylation sites from specialist databases [34-36] (as shown in Figure 4).

We did not perform an equivalent analysis for H/ACA family snoRNAs, since their guide sequences are discontinuous, and therefore non-trivial to predict using the above methods.

### Ancestral state reconstruction

We mapped introns and intronic snoRNAs onto aligned proteins using their respective genomic coordinates. Specifically, the position of the first codon in the exon upstream of the intron in question was converted from genomic coordinates into cDNA coordinates and divided by 3. Introns and intronic snoRNAs located in UTRs were excluded for obvious reasons. All positions were then binned into discrete loci within +/− 5 amino acids, as described previously [77].

The reconstruction of ancestral states requires an underlying phylogeny to establish the timing of emergence or degree of positional conservation of introns, modification sites and snoRNAs. To this end, we created a phylogenetic tree for the 44 species used in our analysis based on the supergroup division suggested by Adl and colleagues [78].

Ancestral states of loci and snoRNA families were reconstructed using Dollo Parsimony as implemented in DolloP from the Phylip package [79]. Parsimony is based on the underlying assumption that presence across taxa is due to common ancestry rather then independent gain and that absence is the default state for any feature [80]. It therefore offers a conservative estimate of the evolutionary processes governing snoRNA distribution. While more complex bayesian implementations are available [81], evolutionary models necessary to accurately describe the dynamics of gain and loss of snoRNAs are, to our knowledge, not yet available. We also note that independent gains, while possible, can often be distinguished, since there appear to be 1) many more sites for insertion than snoRNAs, and 2) snoRNAs from different families inserted into the equivalent position can be readily distinguished. This makes independent gains non-equivalent, in contrast to datasets such as intron insertion at proto-splice sites that may confound simple parsimony [50].

### Placement of SnoRNAs in LECA

For the 22 snoRNA-carrying introns that we were able to trace back to the LECA, we employed three independent criteria to establish homology of the snoRNAs across major taxonomic groups. First, we checked that all putative orthologous snoRNAs belonged to the same Rfam family or clan (where defined). Second, we manually inspected the primary sequence and checked it for conservation of sequence and structural features using the R-coffee algorithm [38] and RNAalifold from the Vienna package [39]. Finally, we examined the predicted site of guide-modification to establish whether each orthlogous group of snoRNAs are also likely to carry out equivalent functions across species. This cross-checking was undertaken to ensure our results were not the result of undetected errors in Rfam; none were detected.

### Host gene expression profile analyses

Expression data from the human transcriptome atlas [45] were obtained from the ArrayExpress Archive ( http://www.ebi.ac.uk/arrayexpress, accession E-TABM-145; [82]. To estimate the global expression level of each gene, we calculated the median array signal for all tissues (removing duplicates, such as brain subsamples). To estimate the expression breadth, we calculated the Shannon entropy as S = −sum (P*i* x ln(P*i*)), where P*i* is the proportion of expression in tissue *i*, and P*i* = E*i*/T; E*i* is the expression of the gene in tissue *i* and T (total expression) is the sum of all expression values for tissues (1, …, *i*).

## Abbreviations

snoRNA, small nucleolar RNA; LECA, Last Eukaryotic Common Ancestor.

## Competing interests

The authors declare that they have no competing interests.

## Authors’ contributions

Conceived and designed analyses: MPH, AMP. Performed analyses: MPH. Analyzed the data: MPH, AMP. Wrote the paper: MPH, AMP. Both authors read and approved the final manuscript.

## Supplementary Material

Additional file 1 Supporting Information. Click here for file

Additional file 2 Table listing mapped snoRNA interactions across SSU/LSU rRNA alignments. Click here for file

Additional file 3 Table listing snoRNA-containing genes traceable to LECA on distribution. Click here for file

Additional file 4 Table of RNA-seq expression data for MRPL3, NDC1 and UBA2PL across Amniotes. Click here for file
