# Supplementary material for: Comparative genomics of eukaryotic small nucleolar RNAs reveals deep evolutionary ancestry amidst ongoing intragenomic mobility
Source: BMC Evol Biol. 2012 Sep 15;12:183. doi: 10.1186/1471-2148-12-183 (PMC3511168; doi:10.1186/1471-2148-12-183)

# Supporting Information for:

**Comparative Genomics of Eukaryotic Small Nucleolar RNAs Reveals Deep Evolutionary Ancestry Amidst Ongoing Intragenomic Mobility**

Marc P. Hoeppner^1^, & Anthony M. Poole^2^

^1^Science for Life Laboratories, Department of Medical Biochemistry and Microbiology, Uppsala University, SE-751 23 Uppsala, Sweden

^2^School of Biological Sciences, University of Canterbury, Private Bag 4800, Christchurch 8140, New Zealand

**This file contains:**

**Table S1.** Rfam families/clans in the eukaryote ancestor

**Table S2.** Clans with multiple LECA_candidate families

**Table S3.** Genomes used in this analysis

**Table S4.** SnoRNA data derived from the literature

**Figure S1**. Conservation of snoRNA-carrying host genes across 44 eukaryote genomes.

**Figure S2.** Conservation of introns in the dataset of 1782 snoRNA-carrying host genes.

**Additional supporting information available online:**

**Additional data file 1.** Mapped snoRNA interactions across SSU/LSU alignments.

**Additional data file 2.** SnoRNA-containing genes traceable to LECA on distribution.

**Additional data file 3.** RNA-seq expression data for MRPL3, NDC1 and UBA2PL across Amniotes.

**Supplementary tables**

**Table S1.** Rfam families/clans in the eukaryote ancestor

| **Rfam acc*** | **Type** | **Common name** | **Comment** |
| --- | --- | --- | --- |
| RF00012 | CD-box | U3 | pre rRNA processing |
| RF00016 | CD-box | SNORD14 | pre rRNA processing |
| RF00069 | CD-box | SNORD24 |  |
| RF00086 | CD-box | SNORD27 |  |
| RF00201 | CD-box | Z278 |  |
| RF00213 | CD-box | R38 |  |
| CL00008 | CD-box | U54 |  |
| CL00038 | HACA-box | SNORA52 |  |
| CL00042 | HACA-box | SNORA65 |  |
| CL00045 | CD-box | SNORD15 |  |
| CL00047 | CD-box | SNORD18 |  |
| CL00049 | CD-box | SNORD25 |  |
| CL00051 | CD-box | SNORD29 |  |
| CL00052 | CD-box | SNORD30 |  |
| CL00053 | CD-box | SNORD31 |  |
| CL00054 | CD-box | SNORD33 |  |
| CL00055 | CD-box | SNORD34 |  |
| CL00056 | CD-box | SNORD35 |  |
| CL00057 | CD-box | SNORD39 |  |
| **CL00059** | **CD-box** | **SNORD43** | Equivalent to archaeal snoRNA sR12 (RF01147)** |
| CL00060 | CD-box | SNORD44 |  |
| CL00061 | CD-box | SNORD46 |  |
| CL00062 | CD-box | SNORD49 |  |
| CL00063 | CD-box | SNORD52 |  |
| CL00065 | CD-box | SNORD59 |  |
| **CL00066** | **CD-box** | **SNORD60** | Equivalent to archaeal snoRNA sR36 (RF01124)** |
| CL00067 | CD-box | SNORD61 |  |
| CL00068 | CD-box | SNORD62 |  |
| CL00069 | CD-box | SNORD74 | also: altern. Splicing |
| CL00070 | CD-box | SORD77 |  |
| CL00071 | CD-box | SNORD88 |  |
| **CL00072** | **CD-box** | **SNORD96** | Equivalent to archaeal snoRNA sR11 (RF01150)** |
| CL00073 | CD-box | SNORD100 |  |
| CL00074 | CD-box | SNORD101 |  |
| CL00076 | CD-box | SNORD110 |  |
| CL00079 | CD-box | snR68 |  |
| CL00080 | CD-box | snoR53 |  |
| CL00081 | CD-box | snoU13 | pre rRNA processing |

*Prefix RF denotes Rfam families, CL denotes multi-family clans.
**Families or clans in bold type carry out modifications that are also conserved in archaea (Gaspin et al., 2000).

**Table S2.** Clans with multiple LECA_candidate families

|  |  | **External Databases** | | | **BLAST map** | | |
| --- | --- | --- | --- | --- | --- | --- | --- |
| **Rfam acc** | **Name** | **Human snoRNA DB** | **Plant snoRNA DB** | **Yeast snoRNA DB** | **Hs** | **At** | **Sc** |
| **SNORD61 clan** | | | | | | | |
| RF00270 | SNORD61 | 18S U1442 | 18S U1381 | 18S 1380 | **X** |  |  |
| RF01170 | U61 | 18S U1442 | 18S U1381 | 18S 1380 |  | **X** |  |
| RF01200 | SNORD125 | 28S U2031  28S A1310  18S C1440 | 25S 1313  25S 657  18S U1379 | 25S 1305  25S 463  18S 1376 | **X** |  |  |
| **SNORD29 clan** | | | | | | | |
| RF00070 | SNORD29 | 28S A4493 | 25S 2936 | 25S 2946 |  |  |  |
| RF00049 | SNORD36 | 18S A668  28S A3703 | 18S 624  25S 2212 | 25S 621  25S 2222 | **X**  **X** | **X** |  |
| RF00212 | SNORD38 | 28S A1858 | 25S 1140 | 25S 1133 | **X** |  |  |
| RF00592 | SNORD78 | 28S G4593 | 25S 3036 | 25S 3047 | **X** |  |  |
| RF01198 | SnoR69Y | 18S 1473 | 18S G1415 | 18S 1412 |  |  |  |
| RF00135 | Z223 | 18S 1197 | 18S 1141 | 18S 1140 |  | **X** |  |
| RF01203 | SnR47 | 18S 669  28S 3706 | 18S 622  25S 2213 | 18S A619  25S A2220 |  |  | **X** |
| RF01302 | SnoU36a | 28S 3708 | 25S A2210  25S G2226 | 25S 2225 |  | **X** |  |
| RF00479 | snR71 | 28S 4540 | 25S 2984 | 25S A2946 |  |  | **X** |
| RF00476 | snR61 | 28S 1858 | 25S 1140 | 25S A1133 |  |  |  |
| RF00475 | SnR69 | 28S 4497 | 25S 2940 | 25S C2948 |  |  | **X** |
| **SNORD33 clan** | | | | | | | |
| RF00532^1^ | Me18S-Um1356 | Nd | Nd | Nd |  |  |  |
| RF00535^1^ | Me28S-Am982 | Nd | Nd | Nd |  |  |  |
| RF00133 | SNORD33 | 18S U1326  28S 1515 | 18S U1270  25S 815 | 18S 1271  25S 810 | **X**  **X** |  |  |
| RF00280 | SNORD51 | 28S A1511 | 25S A814 | 25S 810 |  | **X** |  |
| RF00472 | SnR55 | 18S 1324 | 18S 1268 | 18S U1267 |  |  |  |
| RF00134 | R59 | 28S 1515 | 25S 815 | 25S 810 |  | **X** | **X** |

Underlined entries correspond to previously reported famlies/interactions, grey entries show corresponding sites where no interaction for a given family was found. An X in the respective BLAT-map column indicates sites/interactions recovered by our automated, comparative genomics approach

* Blast-mapped sites that are conserved across 2 or more species

^1^ These snoRNAs are only found in *Drosophila melanogaster*

**Table S3.** Genomes used in this analysis

| Name | Supergroup | Source |
| --- | --- | --- |
| Schizosaccharomyces pombe | Opisthokonts | Ensembl58 |
| Xenopus tropicalis | Opisthokonts | Ensembl58 |
| Anopheles gambiae | Opisthokonts | Ensembl58 |
| Ornithorhynchus anatinus | Opisthokonts | Ensembl58 |
| Monodelphis domestica | Opisthokonts | Ensembl58 |
| Gallus gallus | Opisthokonts | Ensembl58 |
| Ustilago maydis | Opisthokonts | NZ_AACP00000000 |
| Macaca mulatta | Opisthokonts | Ensembl58 |
| Monosiga brevicollis | Opisthokonts | NZ_ABFJ00000000 |
| Anolis carolinensis | Opisthokonts | Ensembl58 |
| Cryptococcus neoformans | Opisthokonts | NZ_ABCN00000000 |
| Drosophila melanogaster | Opisthokonts | Ensembl58 |
| Mus musculus | Opisthokonts | Ensembl58 |
| Danio rerio | Opisthokonts | Ensembl58 |
| Ciona savignyi | Opisthokonts | Ensembl58 |
| Neurospora crassa | Opisthokonts | Ensembl58 |
| Aspergillus nidulans | Opisthokonts | Ensembl58 |
| Pan troglodytes | Opisthokonts | Ensembl58 |
| Caenorhabditis elegans | Opisthokonts | Ensembl58 |
| Homo sapiens | Opisthokonts | Ensembl58 |
| Saccharomyces cerevisiae | Opisthokonts | Ensembl58 |
| Pongo pygmaeus | Opisthokonts | Ensembl58 |
| Debaryomyces hansenii | Opisthokonts | NC_006044-NC_006049,NC_010166 |
| Oryza sativa | Archaeplastida | Ensembl58 |
| Physcomitrella patens | Archaeplastida | NZ_ABEU00000000 |
| Ostreococcus lucimarinus | Archaeplastida | NC_009355-NC_009373 |
| Chlamydomonas reinhardtii | Archaeplastida | NZ_ABCN00000000 |
| Arabidopsis thaliana | Archaeplastida | Ensembl58 |
| Vitis vinifera | Archaeplastida | Ensembl58 |
| Populus trichocarpa | Archaeplastida | NZ_AARH00000000 |
| Tetrahymena thermophila | Chromalveolata | GG662205-GG663362 |
| Phaeodactylum tricornutum | Chromalveolata | Ensembl58 |
| Plasmodium vivax | Chromalveolata | Ensembl58 |
| Plasmodium falciparum | Chromalveolata | Ensembl58 |
| Theileria annulata | Chromalveolata | NC_011098-NC_011100,NC_011129 |
| Thalassiosira pseudonana | Chromalveolata | NZ_AAFD00000000 |
| Toxoplasma gondii | Chromalveolata | ABPA01000000 |
| Entamoeba histolytica | Amoebozoa | DS571162-DS572673 |
| Dictyostelium discoideum | Amoebozoa | Ensembl58 |
| Trypanosoma brucei | Excavata | NZ_AAHB00000000 |
| Naegleria gruberi | Excavata | ACER01000000 |
| Giardia lamblia | Excavata | NZ_AACB00000000 |
| Leishmania major | Excavata | NC_007246-NC_007287 |
| Trichomonas vaginalis | Excavata | NZ_ABFJ00000000 |

**Table S4.** SnoRNA data derived from the literature

| **Species** | **Source** |
| --- | --- |
| *Giardia lamblia* | (Chen et al., 2007) |
| *Plasmodium falciparum* | (Raabe et al., 2010) |
| *Chlamydomonas reinhardtii* | (Chen et al., 2008) |
| *Dictyostelium discoideum* | (Aspegren et al., 2004) |
| *Neuospora crassa* | (Liu et al., 2009) |
| *Gallus gallus* | (Shao et al., 2009) |
| *Arabidopsis thaliana* | (Brown et al., 2003) |
| *Oryza sativa* | (Brown et al., 2003) |

**Supplementary Figures**

**Figure S1**. Conservation of snoRNA-carrying host genes across 44 eukaryote genomes.


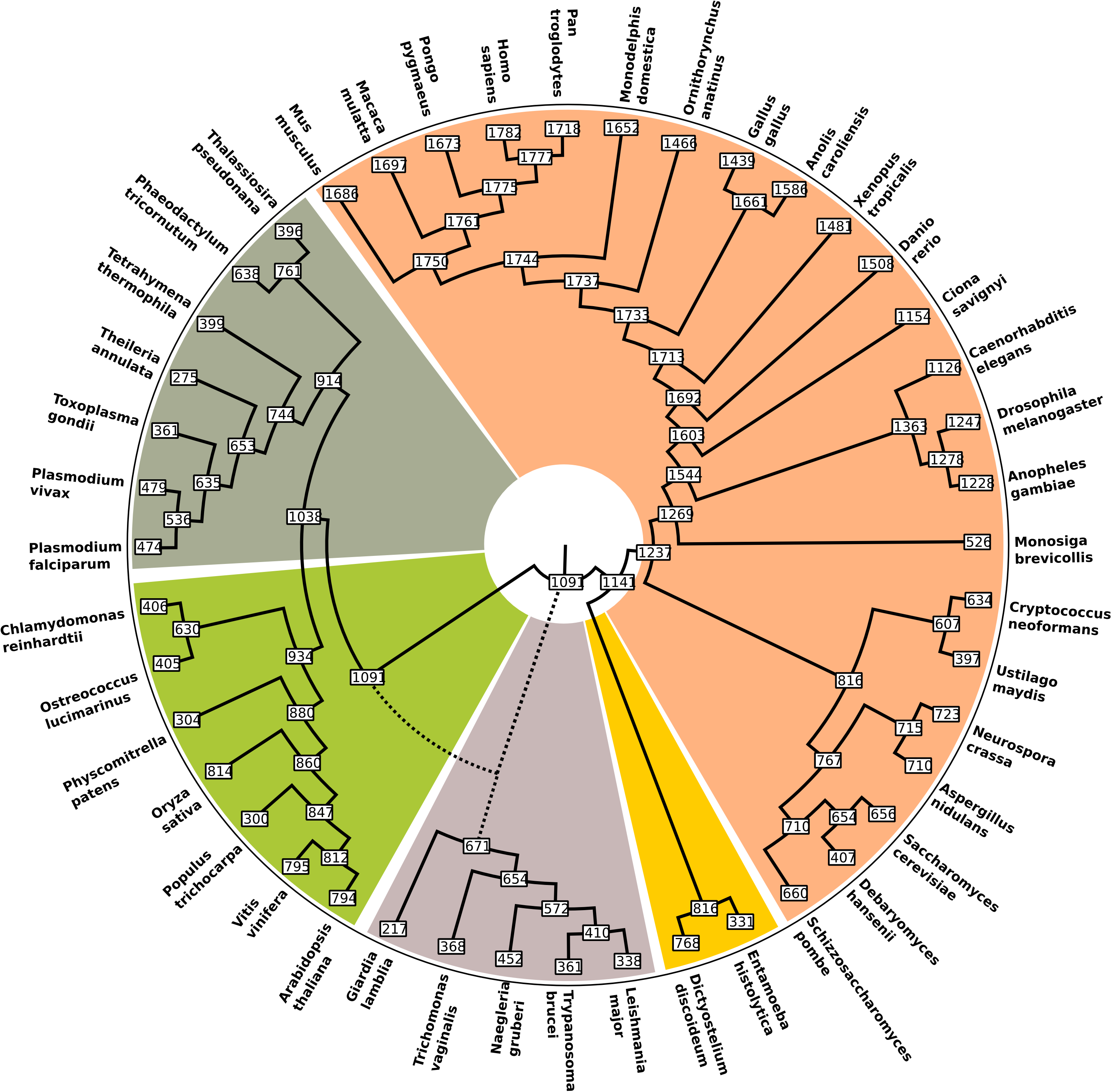


**Figure S2.** Conservation of introns in the dataset of 1782 snoRNA-carrying host genes.


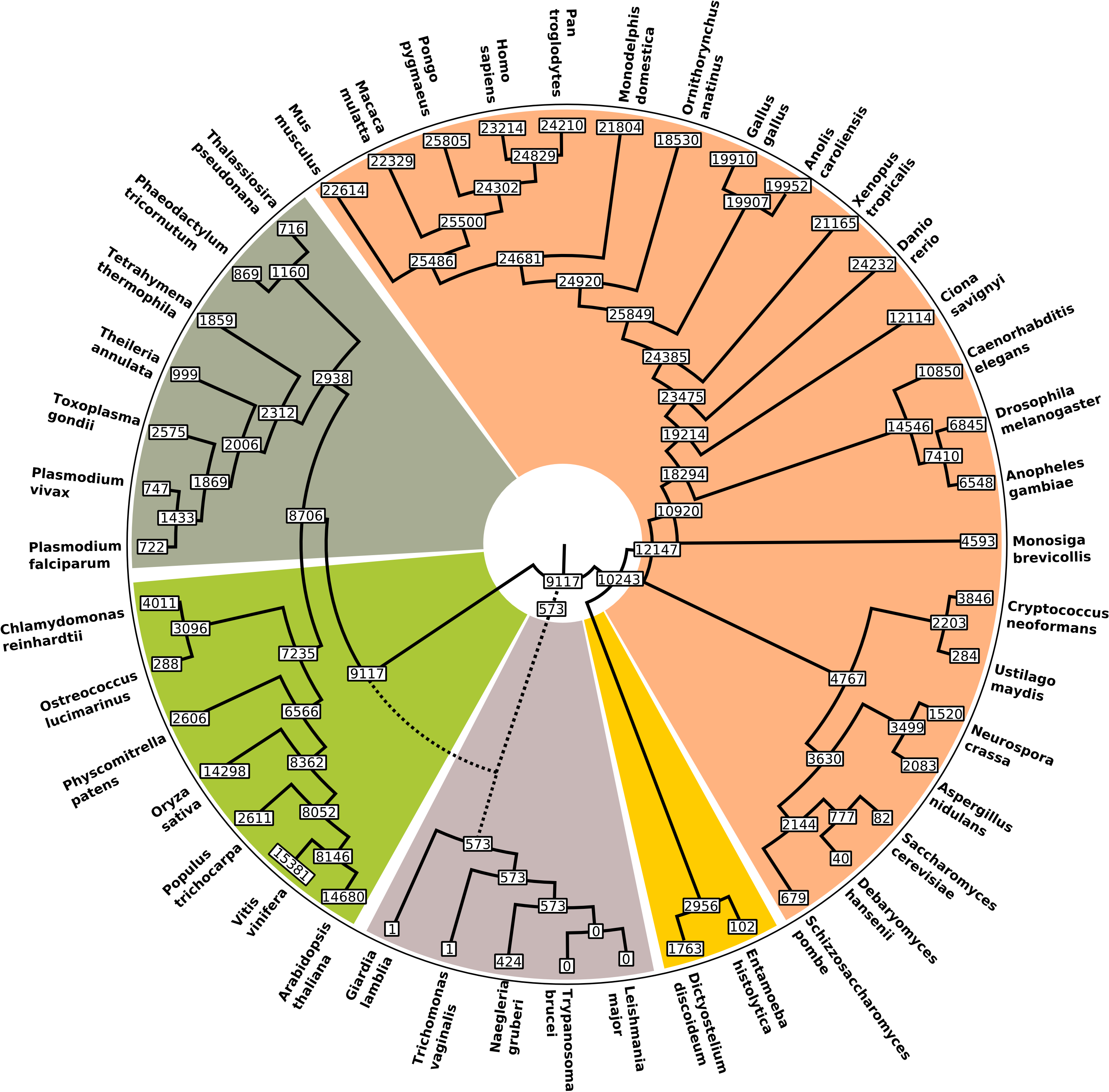

Supplement: Additional file 1 — Supporting Information. [file 1471-2148-12-183-S1.doc]
